# Exercise as a Remedial Agent

**Published:** 1876-03

**Authors:** H. R. Hopkins


ART. II.—*Exercise as a Remedial Agent.* By H. R. Hopkins, M. D.

[Read before the Buffalo Medical Association, Dec. 7, 1875.]

Gentlemen :

My excuse for attempting to interest you for a few moments in the consideration of exercise as a remedial agent, is the fact that from the earliest days of the profession of medicine down to the present time, the profession has exhibited on one hand a tendency to empiricism, and on the other a determination to accomplish results and to seek truth by natural methods—that under the direction of empiricism, investigations of causes or conditions are neglected, and attention is fixed upon finding some external agent which will cure disease, that the empiricism of our forefathers, while not confined to, still finds its chief exponent in the Homeopathy of to-day.—On the other hand that scientific medicine works to-day by the same method adopted by Hippocrates, and seeks through a knowledge of the facts of anatomy, physiology, chemistry, histo-chemistry, and hygiene, to arrive at an intelligent pathology and a successful therapeusis.

That the former believe their successful cases are cured, while the latter see theirs recover—that the tendency of empiricism is to physic giving—while scientific medicine relies more upon the use of natural agents, as rest, exercise, nutrition fresh air and pure water.

That the study of medicine by the scientific method, has produced those glorious results, of which we are most justly proud—and is rapidly developing the crowning achievement of modern civilization, Sanitary Science.

The claims of exercise as a remedical agent, we will consider for the sake of convenience under the following heads :—Natural, Physiological and Chemical.

It will require no labored argument to prove that motion or action is among the distinguishing characteristics of life. That organized creatures are endowed with special apparatuses for executing various motions, that in the head of the animal kingdom this apparatus has reached the greatest state of complexity and perfection, and that his range of possible motions far exceed all others.

Neither is it necessary to more than state the fact that the existence of an organ, includes not only the possibility of its use, but the necessity for that use.

And that an organism can be said to harmonize with its environment only when each separate and particular part of that organism preforms the function for which it was created, and that the Adamic injunction. "By the sweat of thy brow shalt thou eat bread," finds its full interpretation in the revelations of modern science.

Says an eminent medical authority, "Respecting all the organs and functions this is to be observed, that alternate exercise and repose, strengthen them, while a continuance of either enfeebles."

From these axiomatic statements it is easy to deduce the proposition that the daily exercise of our voluntary muscles, to an extent approximating their whole power, is essential to the maintenance of health and a powerful influence in restoration from disease. That exercise stands in the same relation to health as does nutrition to growth.

In the examination of the phyisological relations of exercise, we will, in obedience to its importance, first consider its influence upon the formation of carbonic acid, and the production of animal heat.

In order to make myself intelligible upon this point, it will be necessary to spend a moment in the consideration of the physiological position of carbonic acid.

Authorities all agree in placing carbonic acid at the head of the list of excrementitious products including such substances as urea, cholesterine and creatine, and also agree that it is the great exponent of the vitality or fnnctional activity of the animal, that its production represents both the rate of action of a given organ or tissue and also the rate of nutrition of that tissue, that the force exhibited by an organ as its peculiar function or work, as the contractile force of a muscle, or the formation of gastric juice in the stomach, is the result of the conservation of energy set loose by chemical action, and that carbonic acid, is the most frequent and abundant product of such action, and more than this, that the force of nutrition, by which tissue repair and growth are accomplished, is itself a derivative from this same source of power, the oxidation of the hydrocarbons.

The effect of exercise upon the excretion of carbonic acid has been most accurately observed in man and in animals, and all agree in the conclusion that exercise immensely increases its formation. Flint in his physiology estimates the quantity of oxygen absorbed by muscles at rest and in exercise, as shown by the qnantity of carbonic acid exhaled as from one to two, to one to six according to the intensity of the exercise, and some experiments quoted by Dr. Abbott, in "Sources of Muscular Power," and subsequently referred to by Flint in "Relations of Urea to Exercise," fix the ratio of absorption of oxygen between rest and active exercise as one to seven and eight-tenths, that muscles in active exercise consume nearly eight times the quantity of oxygen they do when at rest.

When we recall that respiration is not performed in the lungs, in the blood, nor yet in the capillaries, but in the innermost parts the organic molecules, of which our tissues are composed, and that respiration is essentially the act of supplying to these molecules the oxygen, which is their life, that respiration is the nutrition of the tissues, and then remember that exercise increases the absorption of oxygen from two to seven-fold, we can no longer withhold from it a chief place among remedial agents.

The influence of exercise upon the production of animal heat is inseparable from its influence upon the production of carbonic acid. I cannot undertake to place before you the history of scientific research, or the theories regarding the causes of animal heat, but will briefly state the views held by physiologists of the present.

That the heat of the body is not formed in the lungs, nor in the liver, nor caused by the friction of the blood, nor by the action of ferments, but as demonstrated by Lavoisier and stated by Papillion, "this heat originates in the totality of those chemical transformations which are going on unceasingly in the depths of the animal organs, and are bringing about the continued renovation of the whole organized substance."

Both physiologists and pathologists are well aware of the importance of maintaining in the body a normal supply of heat and experiment has long since demonstrated, that a loss even of a degree seriously impairs the performance of the vital functions, and has also demonstrated that exercise produces an increase of animal heat, and that in a marked degree.

The manner in which exercise does this we can readily see by its influence upon the formation of carbonic acid.

C. Hanfield Jones reports in the 21st volume of the Transactions of the Royal Society, experiments which he conducted upon thirteen healthy men, and as the result of his observations concludes that moderate exercise raises the temperature of the body in a marked degree. In the cases under his observation the average was 1.8°. The importance of this rise in temperature of the blood cannot be overestimated it having long since been demonstrated, that heat acts as a direct stimulant to all the vital functions, and that a certain temperature is absolutely necessary even for their existence.

Experimenters also agree that long sustained violent exercise is attended with a marked diminution in the quantity of carbonic acid excreted, and a corresponding fall in temperature.

Flint offers this as part explanation for the low temperature he observed in Weston during his five days walk. The average of the five walking pays being two and seven-tenths degrees below the average of the five days before the walk.

The observation on the first day showing a fall of 4.3°, and on the second day a further decline of .5°, the temperature on this day being 94.8° giving for the two days a decline of 4.8°.

Inasmuch as these experiments undertake to have settled some of the most important points in physiological chemistry, they should be subjected to the most rigid scrutiny, and I submit that it is unscientific to accept as the index of the normal relations, between exercise and urea, phenomena which took place in a body of a temperature 2.7° below its normal point, and 1.2° below the limit compatible with health. That it is impossible to arrive at physiological truths from observations upon a body in a pathological condition.

But it is not through experimental or theoretical physiology that exercise as a remedial means will win its way to the regard of our profession; that can only transpire through the concurrence of clinical observation.

This is as it should be. Clinical observation is the way, and the only way, in which the principles derived from physiological, histological and hygienic observations can be verified.

Says Flint, in speaking of the treatment of Pulmonary Tuberculosis, "I rank exercise and out-door life far above any known remedies for the cure of this disease. * * * So deeply impressed am I with the correctness of this view of the regiminal management of disease, that I cannot express myself too emphatically in trying to enforce its practical importance."

Tilt, in Change of Life, claims for exercise "the power of relieving the congestion of internal organs by transferring the blood to the limbs, of depleting the skin by increasing the perspiration, of increasing the excretion of urea, of exhausting those redundant energies which, however little understood, when unemployed produce that sliding scale of phenomena, fidgets, nervousness, temper, hysteria, insanity.

Trousseau, in giving the treatment of diabetes, hepatic colic and gout, says " exercise must be religiously observed, and cannot be too highly recommended."

Bennett, Watson and Aitken all bear testimony to the beneficial effects derived from exercise in the treatment of gouty or scrofulous conditions.

Murchison, in " Functional Derangements of the Liver," finds in lithæmia, a condition, caused by a deficient supply of oxygen to the tissues, as the result of which, the oxidizing processes which go on in the liver and elsewhere are imperfectly performed; causing an increased quantity of lithic acid in the blood; and sees as the result of this condition (lithæmia) numerous diseases, which involve almost every tissue and organ of the body. Murchison recognizes as the cause of this lithæmia, among other elements, insufficient exercise in the open air, which acts chemically, by diminishing the supply of oxygen to the tissues, and mechanically by retarding the circulation of blood through the liver, Bernard having proven that deep inspirations have a powerful influence upon the blood current through the portal vein. This vein having no valves, and being obliged to do the work of an artery with the structure of a vein, is quite dependant upon the suction caused by deep inspirations.

Murchison regards oxygen as the antidote of the *materies morbi* (lithic acid), and places exercise in the open air as a means of pre-eminent importance, both as a therapeutic and as a prophylactic.

Thomas J. Graham, M. R. C. S., in "Treatment of Indigestions and Best Methods of Improving Health," recommends exercise in the open air, in the treatment of dyspeptic, neuralgic, billious and gouty conditions, as a more efficient means than diet or medicine, or both combined.

Samuel Wilks, in the September number of the *London Lancet*, appears in an article full of positive statements bearing upon this question. In reading this article, you are impressed with the idea that he speaks from profund conviction, and not only means what he says, but more than he says. So much does this article please me, it having come to hand after I was well into my reading upon this matter of exercise, that I cannot resist the temptation of quoting the following lines:

"Now, if the question be put thus broadly, Are people suffering from over-work? I, for one, should have no hesitation in saying No. But, on the contrary, if both sexes are taken, I should say the opposite is nearer the truth, and that more persons are suffering from idlenesss than from excessive work. Medically speaking, I see half-a-dozen persons suffering from want of occupation to one who is crippled by his labors.

"I have, then, very little sympathy with the prevalent notion that nervous and other disease are due to overwork. * * * The human body is made for work, physical and mental. The amount of work it can do is of course proportioned to the power of the machine; but, unlike all other machines, its strength is only maintained by use, as assuredly it rusts and decays by disuse. Just as the muscles are better prepared for work by previous training, so the nervous system, whether it be the brain or spinal cord, becomes more energized by use. * * *

"In short, in my experience I see more ailments arise from want of occupation than from overwork; and taking the various kinds of nervous and dyspeptic ailments, which we are constantly treating, I find at least six due to idleness to one from overwork."

Chambers, in his valuable work, "Indigestions," in speaking of what can be done for the treatment of those organic diseases which are usually considered incurable, says, "It is seldom too late to try and administer to the failing organ *the most potent of all remedies*, the human blood of the patient himself made healthy by the means adopted, and flowing in continuously by its own natural channels."

Gentlemen, that sentence contains more wisdom than many whole volumes, and it is in carrying out this thought that exercise becomes valuable. Taking hold as it does of the very foundations of organized life, the use of oxygen in the tissues, we are prepared to find what we do find, that it stimulates and increases, in general and in particular, those functions which in their totality we call nutrition, and when available, should be used in all cases in which defective nutrition exists either as a primary or secondary condition, results having shown that proper exercise exerts a more powerful influence over this condition than any other agent at the command of our profession.

You will pardon me if I go somewhat beyond my subject and add a few lines upon Exercise as a Prophylactic; and if my excuse, that the profession have a tendency to disregard the power of exercise as a remedy is valid, I will add, that as a profession, we are wasting a golden opportunity of demonstrating our familiarity with the principles of preventive medicine, and of serving our patrons, as scientific medical advisers, by failing to impress upon them the power and importance of exercise as a prophylactic. That our profession has failed in this direction, you will appreciate by referring to Flint's chapter upon Prophylaxis, and find that in the enumeration of preventive measures exercise is not even mentioned.

This is in strong contrast with the utterances of the English writers of similar reputation, they seem to be troubled to find terms of sufficient gtrength, in which to exp.ess their appreciation of this element of hygiene. Says John Cheyne, F. R. S., as quoted by Graham, "The weak and valetudinary, the studious and contemplative, ought to make exercise a part of their religion." Says C. Hanfield Jones, "The capacity to resist or endure fatigue well indicates *cœteris paribus* a like power to resist or endure disease well."

History tells us that nations are at first weak and poor, that from their weakness they are obliged to practice industry, frugality and temperance, that through obedience to these hygenic laws they arrive at strength and opulence, that for reason of wealth they forsake the laws of hygiene, and become luxurious, self-indulgent and effeminate, that from this follows loss of strength health and wealth, when the history closes with the disappearance of the people, or they by reason of weakness and poverty return to their obedience to the laws of hygiene.

Does not the history of our own country show us that we are passing through this same scale, that as a people the danger of wealth is upon us, and that the medical profession is the watchman upon the walls, that each indivual member of that profession has a duty he owes to humanity, to his country, to his profession and to himself, to imbue his patrons with the true knowledge of the importance of the daily observance, of the laws of health.

Who can doubt that the influence of Abernethy and Abercrombie, Sir Astly Cooper and other fathers of English medicine, has been a powerful factor in forming the habits of that people, and giving them that taste for out-door exercise which in turn has secured their bodily vigor, and enabled them to resist for centuries, the physically depressing influences of wealth.

In conclusion let us turn from the general and theoretical to the special and practical, and consider a case illustrative of the remedial power of exercise. It is well known to most of you that for the past eight months I have been suffering from a severe lichenous eczema which has resisted the usual means recommended for such troubles. My medical adviser, Dr. Folwell, an expert in diseases of the skin, after some time spent in the investigation of the case, became convinced that the cause of the disease was a fault in assimilation, and this belief was confirmed by an examination of the urine, which was found of a specific gravity of 1030 and loaded with lithic acid. We now congratulated ourselves that the key of the case was well in our hands, and that the condition would soon yield. Accordingly the diet was carefully regulated, tonics Quinine, Arsenic and Strychnine were given and diluents and ant-acids as Muriate of Ammonia, Acetate of Potash, Citrate of Lithia, were persistently exhibited, but withont benefiting the condition which after the second month was associated with an intense stomach indigestion.

The above treatment was supplimented by vapor baths and a week of rest in the country. Of the effects of the latter remedies I have happy recollections.

This treatment extended over the space of four months, at the end of which time my indigestion was confirmed and my skin disease no better. Soon after the beginning of the fifth month my physician being quite out of conceit of medicine, advised me to try the effects of exercise, and I selected as an available means a recent invention the Health Lift, and began exercising at once giving to it half an hour each day, have continued the same up to the present time and with the happiest effect. My indigestion yielded from the first and has not returned; within the first week my urine fell to 1010-1012, with a natural reaction, and that with no other diuretic than a glass of water drank at retiring and on rising; my general feelings have been wonderfully improved and and my skin disease is well, and this notwithstanding, that during this time I have had an increased amount of professional labor and anxiety

From my personal experience and from the result of my observations of its effects upon several of my patients, I have no hesitation in recommending this means of exercise as econmical of time, perfectly under control, and in general, as more scientific than any other at our command.

